# Nirmatrelvir plus ritonavir in patients with underlying rheumatological diseases, in preventing COVID-19 related hospitalization and death

**DOI:** 10.1186/s42358-024-00388-6

**Published:** 2024-07-03

**Authors:** Faiza Javed, Anthony A. Mangino, Paramarajan Piranavan

**Affiliations:** University of Kentucky, Lexington, KY US

**Keywords:** Nirmatrelvir plus ritonavir, COVID-19, Immunosuppression, Rheumatology

The benefits of antiviral therapy in the COVID-19 patient population with underlying rheumatic disease with or without immunosuppression are not entirely clear. The percentage of such patients in a recently published studies (Oral Nirmatrelvir for High-Risk, non-hospitalized Adults with COVID-19) was < 5% [1, 2]. Our study aims to determine the utility of Nirmatrelvir plus ritonavir in patients with rheumatological diseases in preventing COVID-19-associated hospital admissions and mortality. This is a retrospective study in which participants were identified as patients above the age of 18, with a rheumatological condition based on ICD-9 and/or ICD-10 codes who were presented to the University of Kentucky Hospitals between January 1, 2020, and September 30, 2022. The baseline characteristics of patients included in this study are shown in Table 1. Outcomes of interest were in-hospital mortality related to COVID-19 and inpatient or emergency admission due to COVID-19. For both the desired outcomes, two logistic regression models were employed with odds ratios (OR) and 95% confidence intervals obtained for each of two outcomes: In-hospital mortality, and hospitalization or emergency room visits. The first model only considered patients’ Nirmatrelvir plus ritonavir prescription status conditional on their COVID-19 vaccine status. The second model considered patients’ Nirmatrelvir plus ritonavir prescription status conditional on their COVID-19 vaccine status while adjusting for their current prednisone prescription status (used for their rheumatological disease), obesity, and diabetes diagnosis. 2387 patients were reviewed. COVID-19 vaccination was received by 1836 patients (76.9%). Only Nirmatrelvir plus ritonavir prescription was received by 461 patients (18%). Patients who received both COVID-19 vaccination (at least one dose) and Nirmatrelvir plus ritonavir were 86 (3.6%). We did not find any protective effect of nirmatrelvir plus ritonavir among unvaccinated recipients (OR = 1.12; CI:0.86–1.48; *p* = 0.392). Use of nirmatrelvir plus ritonavir among vaccinated patients resulted in a lower likelihood of hospitalization and emergency room visit (OR = 0.46; CI: 0.27–0.78; *p* = 0.004). Another primary outcome considered in the study was death-related/due to COVID-19 patients. Nirmatrelvir plus ritonavir only was not found to be associated with reduced mortality related to COVID-19 (OR = 1.2; CI: 0.65–2.09; *p* = 0.535). All logistic regression results are included in Table 2. This study demonstrates that Nirmatrelvir plus ritonavir provides protection in immunosuppressed patients with rheumatological diseases against severe COVID–19–associated outcomes resulting in emergency room visits and inpatient admission, and significantly in COVID–19 vaccinated individuals. Similar to the study recently published targeting elevated risk individuals [3]. We were not able to observe any significant positive result in the role of Nirmatrelvir plus ritonavir in decreasing COVID-19 mortality unlike the original study [1]. This study has several limitations including small sample size of patients at one institution, impossibility of determining the percentage of each disease, including which predominated, as well as it was not possible to analyze the degree of immunosuppression. More comprehensive studies are needed to study the effect of this drug in such patients.

**Table 1:** Characteristics of the patients at baseline

Characteristics	Untreated patients (*N* = 1926)	Patients treated with Nirmatrelvir plus ritonavir (*N* = 461)	Total patients (*N* = 2387)	*p*-value*
Age				0.798
Mean	52.6 (17.4)	52.4 (18.2)	52.6 (17.6)	
Gender				0.188
Male	633 (32.9%)	167 (36.2%)	800 (33.5%	
Female	1293 (67.1%)	294 (63.8%)	1587 (66.5%)	
Race				0.628
White	1648 (85.6%)	384 (83.3%)	2032 (85.1%)	
African American	237 (12.3%)	69 (15.0%)	306 (12.8%)	
Other	16 (0.8%)	3 (0.7%)	19 (0.8%)	
Diabetes	1290 (67.0%)	323 (70.1%)	1613 (67.6%)	0.224
Obesity	1401 (72.7%)	329 (71.4%)	1730 (72.5%)	0.592
Admission type				0.099
Outpatient	74 (35.0%)	142 (30.8%)	816 (34.2%)	
Inpatient & emergency	1252 (65.0%)	319 (69.2%)	1571 (65.8%)	
COVID-19 vaccination	1461 (75.9%)	375 (81.3%)	1836 (76.9%)	0.014
Rheumatological disease**	2387 (100%)	2387 (100%)	2387 (100%)	NA

*p-values for categorical variables obtained from chi-square tests; *p*-value for Age obtained from Student’s t-test (assuming unequal variances)

**Ankylosing spondylitis, Still’s disease, adult-onset lupus erythematosus, SLE (systemic lupus erythematosus), polymyalgia rheumatica, psoriatic arthritis, psoriatic spondylitis, Reactive arthritis (Reiter’s), R/A w/ rheumatoid arthritis w/o organ or systems involvement, Sjögren’s disease, Wegener’s granulomatosis, Bechet’s syndrome

**Table 2:** Logistic regression results for in-hospital mortality and inpatient/emergency admission

Predictor	Unadjusted model; Odds ratio (95% CI)	Adjusted model; Odds ratio (95% CI)
**In-hospital mortality**		
Nirmatrelvir plus ritonavir only	1.18 (0.65, 2.05)	1.20 (0.65, 2.09)
Covid-19 vaccine & nirmatrelvir plus ritonavir	0.63 (0.10, 2.08)	0.34 (0.05, 1.35)
Covid-19 vaccine only	1.01 (0.56, 1.72)	0.48 (0.18, 1.08)
Prednisone only		0.88 (0.53, 1.43)
Covid-19 vaccine & prednisone		4.43 (1.69, 12.96)**
Obesity		2.11 (1.35, 3.30)**
Diabetes		1.95 (1.24, 3.06)**
**Inpatient or emergency admission**		
Nirmatrelvir plus ritonavir only	1.19 (0.93, 1.54)	1.12 (0.86, 1.48)
Covid-19 vaccine & nirmatrelvir plus ritonavir	0.58 (0.37, 0.90)*	0.46 (0.27, 0.78)**
Covid-19 vaccine only	0.53 (0.43, 0.66)***	0.47 (0.36, 0.61)***
Prednisone only		2.48 (1.98, 3.11)***
Covid-19 vaccine & prednisone		11.02 (6.40, 20.14)***
Obesity		3.74 (2.92, 4.84)***
Diabetes		2.09 (1.68, 2.61)***

*p < 0.05; **p < 0.01; ***p < 0.001

## Data Availability

On request only.
